# Less regurgitation without reverse remodeling: what the PROVE trial teaches us about functional tricuspid regurgitation

**DOI:** 10.1186/s44348-026-00100-w

**Published:** 2026-10-08

**Authors:** Hyun-Jin Kim

**Affiliations:** Division of Cardiology, Department of Internal Medicine, Hanyang University Guri Hospital, Hanyang University College of Medicine, Guri, Korea

**Keywords:** Tricuspid valve insufficiency, Ventricular remodeling, Sodium-glucose transporter 2 inhibitors, Echocardiography, Magnetic resonance imaging

Severe tricuspid regurgitation (TR) is associated with increased mortality, independently of pulmonary pressure and right heart failure [1], yet it remains the least medically addressed among valve lesions. Current guidelines do not provide a class I pharmacologic recommendation for functional TR, offering only a class IIa indication for diuretic therapy in cases of right-sided congestion [2]. While transcatheter repair and replacement can reduce TR grade and improve symptoms, there is no evidence to date of reduced mortality or heart failure hospitalization [3, 4]. Furthermore, all device trials randomize patients receiving an "optimal medical therapy" that lacks a standardized definition.

The PROVE trial, as reported in this issue of the *Journal of Cardiovascular Imaging*, addresses this gap [5]. In this study, 56 patients with significant functional TR and a left ventricular ejection fraction (LVEF) of at least 50% were randomized in a two-by-two factorial design to receive sustained-release carvedilol 16 mg daily, empagliflozin 10 mg daily, both agents, or placebo for 12 months. The primary endpoint, defined as the change in right ventricular (RV) end-systolic volume index measured by cardiac magnetic resonance (CMR), did not differ among the groups. However, empagliflozin reduced vena contracta width (VCW) by 1.45 mm (95% confidence interval [CI], 0.69–2.20), effective regurgitant orifice area by 0.09 cm^2^ (95% CI, 0.02–0.17), and regurgitant volume by 10.7 mL (95% CI, 4.2–17.2) compared to placebo. Thus, improvement was observed at the valve level without corresponding ventricular remodeling.

This dissociation represents the most instructive finding of the trial. The enrolled population had a mean age of 71 years, atrial fibrillation in 89%, an LVEF of 60%, an RV ejection fraction of 48%, and an RV end-systolic volume index of 59 mL/m^2^, a profile consistent with atrial functional TR, in which annular dilation follows right atrial enlargement and leaflet tethering is comparatively modest [6, 7]. The RV in this cohort was dilated, but considerably less so than in the surgical cohort of severe, predominantly ventricular functional TR from which the assumed baseline RV end-systolic volume index of 88.5 mL/m^2^ was taken for the sample size calculation [8]. Enrollment was also halted at 56 of the 180 planned patients because of the COVID-19 pandemic, and 44 patients were analyzed. The trial was therefore underpowered, and the absolute reduction in RV volume that it was designed to detect was one that this phenotype was unlikely to deliver. If the annulus is the primary lesion, RV volume serves only as a distant surrogate for its correction.

The underlying mechanism may therefore be atrial rather than ventricular. Plasma volume contraction and reduced right atrial pressure could narrow the tricuspid annulus and regurgitant orifice without significantly altering RV volumes. The same investigators reported similar findings in the EFFORT trial, where ertugliflozin reduced functional mitral regurgitation without changes in LV volumes or LVEF [9]. However, the PROVE trial cannot confirm this mechanism, as it did not report right atrial volume, annular dimensions, or coaptation gap. Additionally, CMR and echocardiography assessments were separated by a median of 28 days, with only 38% of paired studies conducted on the same day.

For readers focused on imaging, the magnitude of the reported changes requires careful interpretation. A 1.4-mm difference in VCW approaches the reproducibility limits of 2D and proximal isovelocity surface area-based quantification, methods with fragile assumptions in TR [10]. All three echocardiographic endpoints changed in the same direction, which a multiparametric approach considers supportive for severity assessment [10]. These parameters, however, do not contribute equally as independent confirmation. Effective regurgitant orifice area and regurgitant volume are quantitative measures derived from the same proximal isovelocity surface area calculation, with regurgitant volume obtained as the product of effective regurgitant orifice area and the velocity time integral of the TR jet, so the two are mathematically dependent on each other. VCW is a semiquantitative measure taken directly from the two-dimensional color Doppler jet and therefore provides genuinely separate, if less precise, support. All three measures were nonetheless obtained from the same acquisitions by the same reader, so influences acting at the level of image acquisition, including loading conditions and beat selection in atrial fibrillation, apply to each of them. Blinding was also asymmetric at the patient and investigator level. Carvedilol was compared with a matching placebo and proved neutral, whereas empagliflozin was compared with guideline-directed therapy alone and proved positive. Core laboratory readers were masked in both comparisons, which constrains but does not eliminate the influence of unblinded diuretic adjustment on a load-dependent measurement. The carvedilol result, obtained at a dose below heart failure targets and in a rhythm where rate slowing prolongs diastole and augments RV filling, should be interpreted as uninformative rather than negative.

The central hypothesis emerging from the PROVE trial is that, in atrial functional TR, medical therapy may exert its effects primarily on the annulus rather than the ventricle, necessitating the selection of appropriate imaging endpoints. Future trials should prespecify right atrial volume and 3D annular geometry in addition to RV volumes. The ongoing EVENT trial, which randomizes 540 patients with functional TR and preserved ejection fraction to enavogliflozin or placebo with a composite of cardiovascular events or worsening of TR as the primary outcome, will help determine the validity of this signal [5]. In the interim, TR severity should be reassessed after medical therapy has been optimized and before the heart team commits to intervention. The PROVE trial leaves open the possibility that the tricuspid valve is amenable to pharmacologic therapy, and that the effect was sought in the RV when it may reside in the right atrium and the annulus.

## Data Availability

No datasets were generated or analysed during the current study.

## Abbreviations

CI: Confidence interval
CMR: Cardiac magnetic resonance
LV: Left ventricle
LVEF: Left ventricular ejection fraction
RV: Right ventricle
TR: Tricuspid regurgitation
VCW: Vena contracta width
